# Adrenal cortical adenoma-cavernous hemangioma collision tumor: a case report

**DOI:** 10.3389/fonc.2026.1781057

**Published:** 2026-05-22

**Authors:** Yanghong Ou, Lili Wang, Hongguang Qu, Yingmei Jia

**Affiliations:** Department of Radiology, Gansu Provincial Hospital, Lanzhou, China

**Keywords:** adrenal collision tumor, cavernous hemangioma, cortical adenoma, CT, MRI

## Abstract

Adrenal collision tumors (ACTs) are rare entities defined by the coexistence of two histologically distinct, adjacent tumors within a single adrenal gland. Accurate preoperative diagnosis is crucial for management but remains challenging. We present a rare case of ACT in a 49-year-old woman with a three-year history of limb weakness. Imaging revealed a well-defined right adrenal mass with two distinct components. On detailed multiplanar reconstruction, the cortical adenoma component was located in the lateral (outer) portion of the right adrenal gland, occupying the superior-posterior aspect and abutting the perirenal fat (approximately 35% of total tumor volume). The cavernous hemangioma component was located in the medial (inner) portion, occupying the central and inferior-anterior aspect adjacent to the inferior vena cava (approximately 65% of total tumor volume). The two components met at a well-defined planar interface oriented obliquely from superolateral to inferomedial, without evidence of infiltration. CT and MRI demonstrated distinct imaging characteristics for each component. Pathological examination confirmed the diagnosis, demonstrating a cortical adenoma coexisting with a cavernous hemangioma, sharply demarcated by a fibrous septum. This case underscores the importance of recognizing the characteristic imaging patterns of different tumor components within a single adrenal lesion. When atypical features are present within an otherwise typical tumor, an ACT should be considered to guide appropriate clinical management.

## 1 Introduction

An adrenal collision tumor (ACT) is a relatively rare entity characterized by the coexistence of two histologically distinct tumors abutting each other within the same adrenal gland. Establishing a definitive diagnosis is challenging but critical, as it may affect tumor staging, management and prognosis, particularly when one component represents metastatic disease. CT and MRI studies play an important role in the detection, characterization and follow-up of ACTs.

In this study, we retrospectively analyzed the CT, and MRI findings, pathological features and clinical characteristics of a case of ACT to enhance understanding and recognition of this rare condition.

## 2 Case presentation

A 49-year-old woman presented with a three-year history of intermittent and progressive limb weakness/discomfort over 3 years. Imaging revealed a well-defined right adrenal mass. She denied a history of hypertension, coronary heart disease, diabetes mellitus, or any relevant family history. Physical examination revealed normal muscle strength and deep tendon reflexes in all extremities, with no sensory deficits. Given the nonspecific symptoms, initial laboratory evaluation was performed, revealing normal angiotensin II, cortisol, adrenocorticotropic hormone (ACTH), urinary vanillylmandelic acid (VMA) and aldosterone/renin ratio (**Table 1**).

**Table 1:** Initial laboratory findings.

Parameter	Measured value	Unit	Normal range	Interpretation
Angiotensin II	53.3	ng/ml	16.2–64.2	Normal
Cortisol	200.9	nmol/l	102–536	Normal
ACTH	23.2	pg/ml	7.2–63.3	Normal
Urinary VMA	13.0	μmol/24h	0–68.6	Normal
Aldosterone	14.1	ng/dl	5.9–17.4	Normal
Renin	0.14	ng/ml/h	0.05–0.79	Normal

ACTH, adrenocorticotropic hormone; VMA, vanillylmandelic acid.

### 2.1 Imaging findings

The patient initially underwent chest-abdomen CT (non-contrast) as part of the workup for limb weakness, which incidentally revealed a right adrenal mass. No other initial imaging was performed as the clinical presentation did not suggest central nervous system or spinal pathology (normal neurological examination, no bowel/bladder dysfunction, normal lower limb reflexes). Subsequent imaging was protocol-driven and focused entirely on adrenal characterization: enhanced CT of upper abdomen, followed by adrenal MRI with chemical shift imaging for lipid quantification and lesion characterization.

Adrenal CT imaging demonstrated a well-defined, oval mass measuring approximately 60 mm × 50 mm × 35 mm in the right adrenal gland. The lesion exhibited heterogeneous attenuation.

On detailed multiplanar reconstruction, the mass was found to consist of two distinct components with separate measurements and anatomical locations. The cortical adenoma component measured 2.5 cm × 2.0 cm × 1.8 cm and was located in the lateral (outer) portion of the right adrenal gland, occupying the superior-posterior aspect of the mass and abutting the perirenal fat. This component accounted for approximately 35% of the total tumor volume. The cavernous hemangioma component measured 4.0 cm × 3.5 cm × 3.0 cm (including the cystic cavity) and was located in the medial (inner) portion of the gland, occupying the central and inferior-anterior aspect, immediately adjacent to the inferior vena cava. This component accounted for approximately 65% of the total tumor volume. The two components met at a well-defined planar interface oriented obliquely from superolateral to inferomedial, measuring approximately 1–2 mm in thickness on imaging, without evidence of infiltration or tissue mixing.

**Figure 1:**
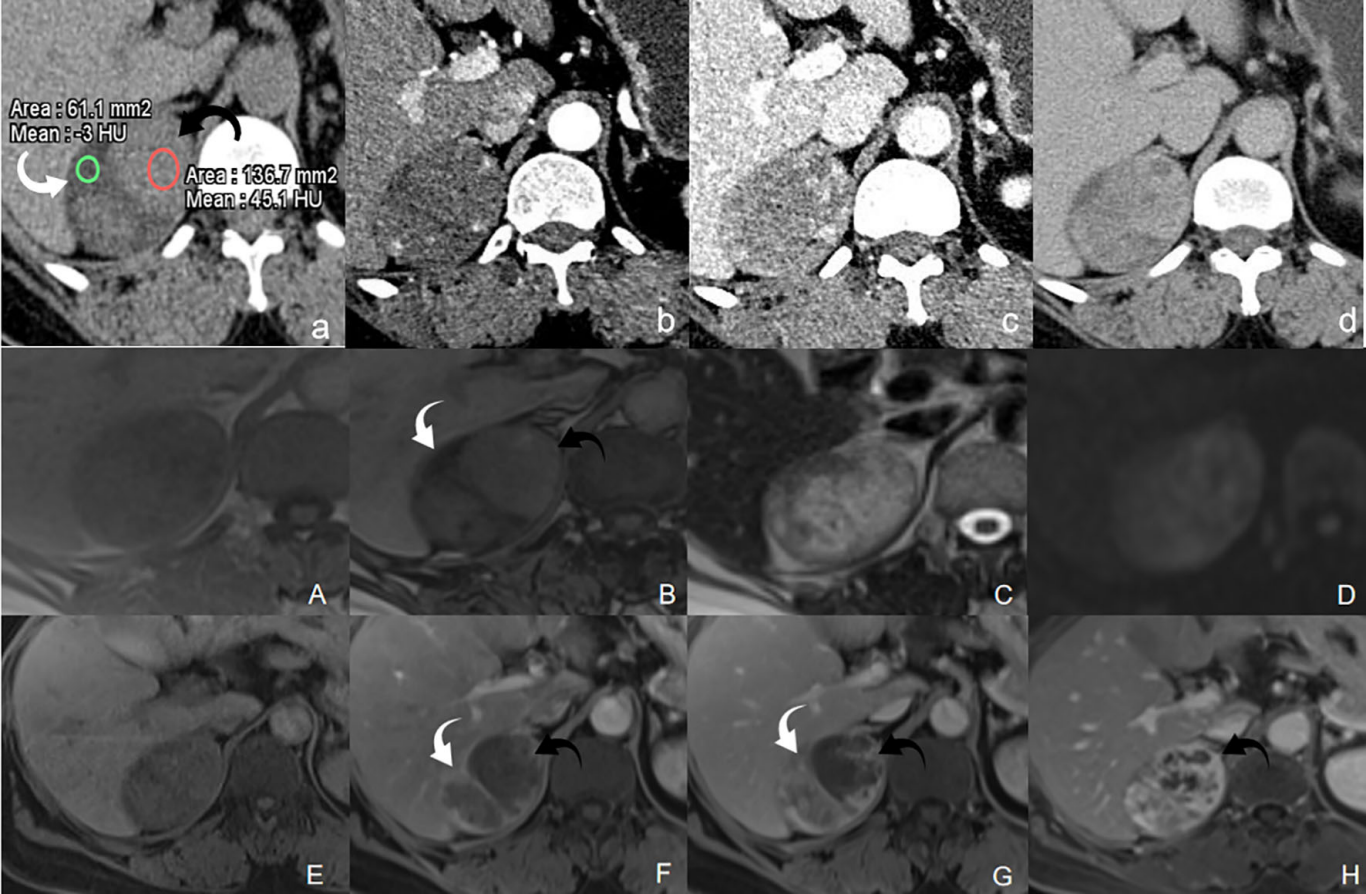
CT scan shows a well-defined, oval mass in the right adrenal gland. **(A)** The mass showed heterogeneous density: the hyperdense area had a CT value of 36~44.4 HU (black arrows), while the hypodense area measured -4.6~-2.4 HU (white arrows). **(B-D)** On enhanced imaging, the hyperdense area exhibited persistent progressive enhancement, whereas the hypodense area showed mild arterial-phase enhancement, increased venous-phase enhancement, and delayed-phase washout.

The adenoma component showed hypoattenuation with a mean CT value of -4.6~-2.4 HU (indicating lipid-rich content), whereas the hemangioma component showed hyperattenuation with a mean CT value of 36~44.4 HU. On contrast-enhanced CT, the hemangioma component showed progressive and persistent enhancement, whereas the adenoma component showed mild enhancement in the arterial phase, increased enhancement in the venous phase, and washout in the delayed phase (**Figure 1**).

On MRI, the right adrenal mass demonstrated heterogeneous signal intensity, with distinct areas of loss of signal intensity (indicating intracellular lipid) and signal hyperintensity on opposed phase (OP) imaging. On T2-weighted imaging, the lesion showed mixed intensity, with hyperintense areas corresponding to those with high signal intensity on OP imaging. Diffusion-weighted imaging revealed no evidence of restricted diffusion. On contrast-enhanced MRI, the areas with signal loss on OP imaging exhibited rapid washout, whereas the hyperintense areas showed heterogeneous, persistent, and progressive enhancement (**Figure 1**).

On T1-weighted in-phase/opposed-phase imaging (A, B), distinct signal reduction (white arrows) and elevation areas (black arrows) were noted. T2-weighted imaging (C) revealed mixed signals, with high signal intensity in the opposed-phase elevated areas. Diffusion-weighted imaging (D) showed no restricted diffusion. Enhanced MRI (E-H) demonstrated rapid washout in the opposed-phase reduced areas (white arrows) and heterogeneous persistent progressive enhancement in the opposed-phase elevated areas (black arrows).

### 2.2 Therapeutic intervention

Given the tumor size (>4 cm), and inability to exclude malignancy preoperatively, surgical resection was indicated. After multidisciplinary discussion and informed consent, the patient underwent laparoscopic right adrenalectomy via a retroperitoneal approach. Intraoperative findings confirmed the presence of two distinct components with a clear demarcation. No intraoperative complications occurred.

### 2.3 Pathologic examination

**Figure 2:**
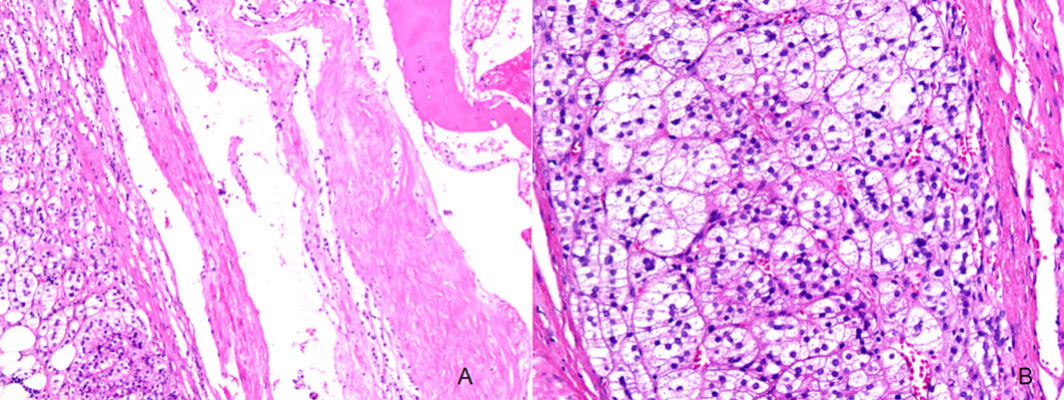
Histopathologic findings of the right adrenal gland tumor. **(A, B)** Hematoxylin and eosin staining (100×, 200×) shows a 4 cm cystic cavity on section. The cyst was well-demarcated from surrounding tissue, accompanied by abundant thin-walled blood vessels and localized thrombus formation with organization.

Gross examination of the resected right adrenal gland confirmed the imaging findings. The specimen contained a yellowish, encapsulated, solid nodule measuring 2.5 cm × 2.0 cm × 1.8 cm in the lateral portion, corresponding to the cortical adenoma. Adjacent to this, in the medial portion, was a dark red, spongy, cystic lesion measuring 4.0 cm × 3.5 cm × 3.0 cm, corresponding to the cavernous hemangioma. The two components were sharply demarcated by a fibrous septum approximately 2 mm thick, without any areas of transition or admixture. A thin rim of compressed, normal-appearing adrenal cortex (2–3 mm thick) was present at the peripheral margin of the adenoma component, distinguished by its brown color and thin, cortical layer morphology (**Figure 2**).

Microscopically, the adenoma component consisted of cells arranged in nests and cords with clear, lipid-rich cytoplasm and small, regular nuclei, consistent with adrenal cortical adenoma. The hemangioma component showed abundant thin-walled vascular spaces containing blood and organized thrombus, consistent with cavernous hemangioma. The fibrous septum between the two lesions contained dense collagen fibers without any transitional tissue.

Immunohistochemical analysis showed distinct profiles for each component:

Adenoma component: Melan-A(+), α-inhibin(+), CgA(-), Syn(-), CK8/18(+), Vimentin(-).

Hemangioma component: CD31(+, vascular endothelial), CD34(+, vascular endothelial), Vimentin(+), CK8/18(-), Melan-A(-), α-inhibin(-).

The Ki-67 proliferation index was 3% in the adenoma component and <1% in the hemangioma component. Based on the imaging, pathological and immunohistochemical findings, a diagnosis of adrenal cortical adenoma coexisting with cavernous hemangioma, an adrenal collision tumor (ACT), was established (**Table 2**).

**Table 2:** Comparison of imaging and pathological features between the two components of the adrenal collision tumor.

Characteristics	Adrenal cortical adenoma component	Cavernous hemangioma component
Size	2.5 cm × 2.0 cm × 1.8 cm	4.0 cm × 3.5 cm × 3.0 cm
Location	Lateral (outer) portion	Medial (inner) portion
CT value	-4.6 to -2.4 HU (lipid-rich)	36-44.4 HU
CT enhancement	Mild arterial enhancement, venous increase, delayed washout	progressive enhancement
MRI chemical shift	signal drop on opposed-phase	No signal drop on opposed-phase
T2-weighted MRI	Intermediate signal	hyperintense
DWI	No restricted diffusion	No restricted diffusion
MRI enhancement	Rapid washout	progressive enhancement
Gross pathology	Yellow, homogeneous, solid	Dark red, spongy, cystic lesion

DWI, diffusion-weighted imaging; HU, Hounsfield units.

### 2.4 Follow-up and outcomes

The patient recovered uneventfully. Limb weakness markedly improved within one week postoperatively and completely resolved after one month. At 6-month and 12-month follow-up visits (September 2024 and March 2025), the patient remained symptom-free with no evidence of recurrence on CT imaging. The contralateral left adrenal gland appeared normal in morphology and function. Laboratory evaluation showed normal adrenal hormone levels (cortisol, aldosterone, androgens) at all follow-up visits, with no requirement for hormone replacement therapy. No surgery-related complications (infection, bleeding, adrenal insufficiency) were observed.

## 3 Discussion

Adrenal collision tumors (ACT) refer to coexistence of two contiguous but histologically different tumors within the same adrenal gland. These tumors may comprise two benign tumors, two malignant tumors, or a combination of benign and malignant tumors but they differ from composite tumor in their histogenesis (1, 2).

Based on our literature review of PubMed database (through 2025), approximately 36 cases of adrenal collision tumors have been reported in English literature. The most common combination is adenoma with metastasis (14 cases, approximately 38.9%, most commonly lung, renal cell, and breast carcinoma) (2–6). Rare combinations (<5% each) include adenoma with hemangioma, ganglioneuroma with paraganglioma, and myelolipoma with schwannoma (7–11).

The combination of cortical adenoma with cavernous hemangioma is exceedingly rare, with only 3 cases previously reported in PubMed-indexed literature (7, 8). Our case represents the fourth reported case and the most comprehensively characterized in terms of imaging-pathological correlation and long-term follow-up.

The pathogenesis of ACT remains debated. Fujii H et al. (12) proposed three hypotheses regarding the formation of collision tumors: (1) independent clonal development of two distinct tumors; (2) a single tumor clone with biphenotypic genetic expression leading to divergent histological differentiation; and (3) genetic heterogeneity within a tumor clone resulting in two histologically distinct subclones. Schwartz LH et al. (13) suggested that the high prevalence of adrenal adenomas and metastases, combined with the small size of the adrenal gland, increases the likelihood of concurrent lesions occurring within the same gland. In our case, the sharp demarcation without transitional tissue supports the independent origin hypothesis.

Accurate preoperative diagnosis of ACT is essential for several reasons: (1) guiding personalized treatment strategies; (2) facilitating comprehensive pathological sampling to avoid misdiagnosis; and (3) enabling accurate radiologic–pathologic–clinical correlation.

Multiphase CT and MRI with chemical shift imaging are crucial. In this case, distinct imaging characteristics of each component enabled preoperative suspicion of ACT: lipid-rich adenoma (low attenuation, signal drop on opposed-phase, rapid washout) versus hemangioma (progressive persistent enhancement, T2 hyperintensity).

The primary challenge is recognizing that two distinct entities coexist rather than a single tumor with atypical features. If attention focuses only on typical adenoma characteristics while ignoring the atypical enhancement area, the collision tumor may be missed, leading to incomplete resection or inadequate sampling.

The differential diagnoses for adrenal collision tumors include adrenal adenoma with hemorrhage, adrenocortical carcinoma, adrenal metastasis, and pheochromocytoma. Hemorrhage into adenoma lacks the clear interface and opposed-phase signal characteristics of collision tumors. Cortical carcinoma typically exceeds 6 cm with irregular, invasive borders and necrosis, unlike the well-defined margins here. Metastases occur in patients with known malignancy, show no lipid content or signal drop, and are often bilateral. Pheochromocytoma presents with typical symptoms, marked T2 hyperintensity, and intense enhancement, distinct from collision tumor features.

Benign-benign combinations (as in this case) carry excellent prognosis with surgical cure. Benign-malignant combinations require staging and treatment based on the malignant component.

This case offers complete imaging-pathology correlation, a rare benign-benign combination enriching literature, detailed immunohistochemical confirmation, and excellent follow-up outcomes. Limitations include single-case design without statistical power, absence of genetic testing for clonal analysis, and lack of preoperative biopsy since collision tumor was unsuspected.

In conclusion, adrenal collision tumors exhibit characteristic imaging and pathological features. Recognition of distinct tumor components and their corresponding imaging patterns is essential for accurate preoperative diagnosis and appropriate clinical management.

## 4 Patient perspective

Limb weakness markedly improved within one week postoperatively and completely resolved after one month. At 6-month and 12-month follow-up visits (September 2024 and March 2025), the patient remained symptom-free with no evidence of recurrence on CT imaging. The contralateral left adrenal gland appeared normal in morphology and function. Laboratory evaluation showed normal adrenal hormone levels (cortisol, aldosterone, androgens) at all follow-up visits, with no requirement for hormone replacement therapy. The patient stated that the laparoscopic surgery recovery was very quick, and I could resume normal activities within a week.

## Data Availability

The original contributions presented in the study are included in the article/Supplementary Material. Further inquiries can be directed to the corresponding author.
